# Influence of pH Regulation Mode in Glucose Fermentation on Product Selection and Process Stability

**DOI:** 10.3390/microorganisms4010002

**Published:** 2016-01-04

**Authors:** Zuhaida Mohd-Zaki, Juan R. Bastidas-Oyanedel, Yang Lu, Robert Hoelzle, Steven Pratt, Fran R. Slater, Damien J. Batstone

**Affiliations:** 1 Faculty of Civil Engineering, Universiti Teknologi MARA (Pulau Pinang), 13500 Permatang Pauh, Pulau Pinang, Malaysia; cezuhaida@ppinang.uitm.edu.my; 2 Institute Center for Energy (iEnergy), Masdar Institute of Science and Technology, 54224 Abu Dhabi, United Arab Emirates; jbastidas@masdar.ac.ae; 3 Advanced Water Management Centre, The University of Queensland, St. Lucia, QLD 4072, Australia; y.lu@awmc.uq.edu.au (Y.L.); r.hoelzle@awmc.uq.edu.au (R.H.); s.pratt@uq.edu.au (S.P.); frances.r.slater@gmail.com (F.R.S.)

**Keywords:** fermentation, mixed culture, glucose, pH control, pH regulation method

## Abstract

Mixed culture anaerobic fermentation generates a wide range of products from simple sugars, and is potentially an effective process for producing renewable commodity chemicals. However it is difficult to predict product spectrum, and to control the process. One of the key control handles is pH, but the response is commonly dependent on culture history. In this work, we assess the impact of pH regulation mode on the product spectrum. Two regulation modes were applied: in the first, pH was adjusted from 4.5 to 8.5 in progressive steps of 0.5 and in the second, covered the same pH range, but the pH was reset to 5.5 before each change. Acetate, butyrate, and ethanol were produced throughout all pH ranges, but there was a shift from butyrate at pH < 6.5 to ethanol at pH > 6.5, as well as a strong and consistent shift from hydrogen to formate as pH increased. Microbial analysis indicated that progressive pH resulted in dominance by *Klebsiella*, while reset pH resulted in a bias towards *Clostridium* spp., particularly at low pH, with higher variance in community between different pH levels. Reset pH was more responsive to changes in pH, and analysis of Gibbs free energy indicated that the reset pH experiments operated closer to thermodynamic equilibrium, particularly with respect to the formate/hydrogen balance. This may indicate that periodically resetting pH conforms better to thermodynamic expectations.

## 1. Introduction

Fermentation is commonly used to produce food materials (beverages, dairy products), renewable fuels (hydrogen, ethanol), pharmaceuticals (antibiotics), and industrial chemicals (acetate, butyrate). In industrial fermentation, specialized pure microbial cultures are normally used to generate specific products. This requires expensive, sterile production conditions with high-quality raw materials. In contrast, mixed culture fermentation (MCF) uses environmentally ubiquitous organisms to produce a mixture of products depending on the environmental conditions [1,2]. As they originate from the environment, mixed cultures do not require expensive culture maintenance. In addition, they are capable of growing on a mixture of substrates of variable composition and non-sterile substrates [3,4]. Because of this, mixed culture has the potential to reduce costs, increase reactor loading rates, and allow for continuous reactors, as opposed to batch operation [2,3]. The premise is that one can continuously manipulate product mixtures by changing operational conditions [2]. The key limitation to industrial implementation of MCF is that the product spectrum often varies from thermodynamic expectations although the system is systematic and repeatable [5]. This mainly occurs due to a lack of understanding of how operational factors affect the microbial community/functionality, and hence the product spectrum.

pH is often considered the most important regulating factor in glucose fermentation [5,6,7,8]. Changes in proton availability influence reductase activity, and hence intracellular and extracellular microbial activity [9]. Extensive studies have been conducted to optimize MCF production towards specific products from glucose including: ethanol [10], specific organic acids [5,8], or hydrogen [11], with controversial results. For example, Ren *et al.* [10] found that ethanol production was maximized when pH values were between 4.3 and 4.9 (yield: 0.4–0.9 mol ethanol per mol glucose), whilst Temudo *et al.* [5,12] found that ethanol production was maximized at pH between 6.25 and 8.5 (yield: 0.58–0.7 mol ethanol per mol glucose). The role of pH in regulating product spectrum has also been studied using pure cultures, more often of *Clostridium* spp. In a study conducted with *Clostridium pasteurianum*, Heyndrickx *et al.* [13] found that ethanol was formed at the level of 0.02 mol per mol of consumed glucose when pH was regulated at 5.5 and the yield increased three times at pH 8.0. Other studies found that pH values affected the gas and microbial metabolite production when feeding glucose [14] or alanine [15]. Butyric acid fermentation of xylose by *C. tyrobutyricum* changed from predominant butyric acid at pH 6 to lactate and acetate at pH 5 [16].

Microbial community structure in MCF is also influenced by pH, based on the scarce data in the literature. Temudo *et al.* [12] used denaturing gradient gel electrophoresis (DGGE) to find that *Clostridium* dominated at high (7.5–8.5) and low (4.0–5.5) pH, while *Klebsiella* dominated at intermediate pH levels (6.25–7.0). A wide range of organisms are capable of fermenting glucose to organic acids and the microbial diversity will be increased when complex feedstocks are used (e.g., mixed waste). In this case, variation in product yield and spectrum is expected [17].

Fermentation has been classified in three broad types of reactions; namely (1) butyrate-type; (2) propionate-type; and (3) ethanol-type fermentations. Acetate is always produced as a volatile fatty acid (VFA) product. Excess carbon and electrons are released as carbon-dioxide and hydrogen respectively. In butyrate type fermentation, butyrate and acetate are produced as main end products and hydrogen and carbon dioxide are produced as by-products [18]. In propionate type fermentation, propionate and acetate are produced as main products, valerate is produced in small amounts and there is no substantial gas production [19]. In ethanol-type fermentation, ethanol and acetate are produced as main end products and hydrogen and carbon dioxide are produced as by-products [19].

The effect of pH on regulation of these pathways is understood in broad terms, but there are unexplained deviations [6,7]. For example, it is unknown whether product spectrum is dominated by phylogenetic (microbial community) or physiological (chemical) factors. When pH is changed incrementally and progressively, the culture gradually adapts to a new pH and is acclimatized. This adaptation results in a gradual change in product mix as pH changes, with particular shifts to propionate and/or ethanol at high and low pH values [8,11,20,21]. The alternative is to set a reference pH in advance and then adjust to another pH (reset). Temudo *et al.* [5] and Van Ginkel and Logan [21] observed a rapid change in product mixtures when pH was changed in this way. At first, butyrate yield was high (yield: 0.5–0.7 mol per mol·glucose) when pH was between 4 and 5.5. A sudden “swap” between butyrate and ethanol production occurred at pH 6 and ethanol yield was high (yield: 0.6–0.7 mol per mol·glucose) at pH between 6 and 8.5. In the same way, hydrogen yield was between 1.2 and 1.6 mol per mol·glucose when pH was between 4 and 6.5. A sudden decrease of hydrogen yield (0.1–0.4 mol per mol·glucose) was observed when pH was between 7 and 8.5 [5]. 

One of the key questions is whether mixed cultures behave differently when the pH is gradually altered or undergoes large step changes. This paper evaluates comparative product spectrum when pH is step changed from a central value versus gradually changed.

## 2. Experimental Methods

### 2.1. Inoculum

Inoculum was 200 mL anaerobic digestate from a full-scale mesophilic anaerobic digester in Brisbane, Australia and the reactor was re-inoculated before each experiment. Reactors were inoculated in batch mode at 35 °C, without pH regulation for a week. The continuous flow was initiated once gas production was observed.

### 2.2. Reactor Setup

**Figure 1:**
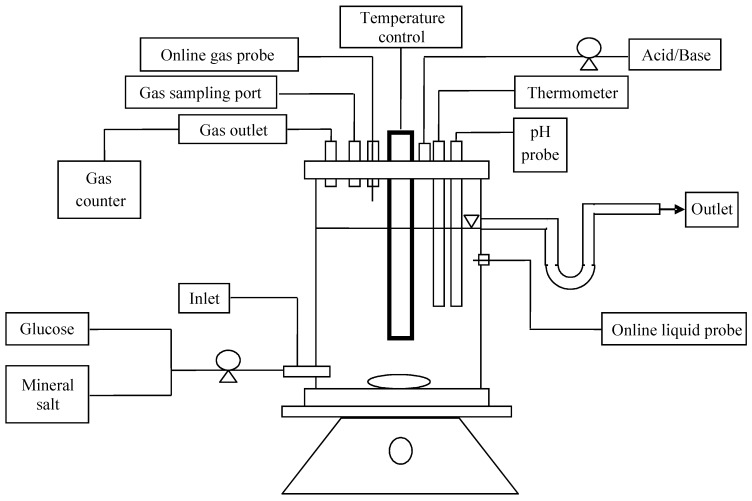
Schematic diagram of reactor set-up.

A 1.5 L continuous stirred tank reactor (CSTR) (1.3 L working volume) was used. Prior to starting each experiment, the reactor was thoroughly cleaned and sparged with N_2_ gas before inoculation. The reactor was equipped with an immersed glass heater (25W Aqua One^TM^, Southampton, UK) to maintain the temperature at 35 °C and a pH probe to control the pH by feeding 1 M NaOH by peristaltic pump. A glass thermometer was added to monitor the temperature. The system was fed by a peristaltic pump (Watson-Marlow Inc., Wilmington, MA, USA) from separate containers of pure glucose solution and basal media. A glass U-tube was fixed to the effluent port to provide a liquid lock, and hence anaerobic conditions. Gas flow was measured by a tipping-bucket type meter, with a bucket volume of 3 mL, and a constant pressure of approx. 3 cm water. All equipment was interfaced to computers via a programmable logic controller (and interfacing software used for data logging and set-point modification. The set-up is illustrated in Figure 1.

### 2.3. Reactor Operation

Three series of experiments were done in a CSTR with a feed concentration of 5 g/L glucose in basal anaerobic media [22] at 35 °C, with a 6 h hydraulic retention time (HRT) and pH varying from 4.5 to 8.5 ( 8.0 in the third series as the culture failed at 8.5). During the progressive pH approach, pH was stepped progressively from pH 5.5 up to pH 8.0, then from pH 5.5 to pH 4.5; with a pH interval of 0.5. The actual points were: 5.5 → 6.0 → 6.5 → 7.0 → 7.5 → 8.0 → 8.5 → 5.5 → 5.0 → 4.5, with steady state reached and held for 12 h (2 HRTs) at each pH. The entire cycle was done twice, and results presented here are the average from the two replicate experiments. Errors represent variation in replicate samples and experiments.

During the reset pH approach, pH was changed from pH 5.5 to the next pH with a 0.5 interval, then reset back to pH 5.5. The pH was reset according to this order: 5.5 → 5.0 → 5.5 → 4.5 → 5.5 → 6.0 → 5.5 → 6.5 → 5.5 → 7.0 → 5.5 → 7.5 → 5.5 → 8.0 → 5.5. The pH extrema of 4.5 and 8.0 or 8.5 were the points at which washout occurred. The central pH of 5.5 was chosen as the native pH with no acid or base dosing.

Reactor performance was monitored by membrane inlet mass spectrometer (MIIMS) [22] online with separate MIMS probes in liquid and gas phases. The system was considered to be in steady-state when the signals from MIMS were stable.

### 2.4. Chemical Analysis

**Gas composition.** Gas composition was analyzed using a gas chromatograph (Shimadzu GC-8A, Brisbane, Australia) equipped with a thermal conductivity detector. The content of hydrogen (H_2_) and carbon dioxide (CO_2_) were measured on a daily basis.

**Liquid sample.** Five liquid samples from the reactor and two liquid samples from the inlet were collected over two HRT at steady state. VFAs, glucose, lactate, formate, pyruvate, and ethanol were measured by GC-FID (PerkinElmer, Melbourne, Australia) with polar capillary column (Agilent Technologies, Santa Clara, CA, USA) at 140 °C. Samples were preserved with 0.05% of sodium azide prior to measurement to avoid further degradation of substrate. 100 mL of unfiltered sample were collected from the outlet port for total solid (TS) and volatile solid (VS) determination as described in standard methods [23].

### 2.5. Data Processing and Statistical Analysis

Chemical oxygen demand (COD) equivalent calculations were done on the stoichiometric oxygen requirement of the pure compound (excluding ammonia). For example, for acetate:

CH_3_COOH + 2O_2_ → 2CO_2_ + 2H_2_O (64 gCOD/mole·Acetate)

Errors in mean concentrations were estimated from a two tailed *t*-test:

(1) E95=sxtn−1,α/2n

where *s_x_* is the standard deviation of the replicates, *n* is the number of replicates (generally 5), *t_n-1,__α/2_* is the *t_crit_* for *n-1* degrees of freedom, and *α*/2 = 0.025 (5% significance threshold) (*t_4,0.025_* = 2.776). Errors in calculated values were determined through analytical propagation of variance [24].

### 2.6. Microbial Analysis

Microbial analysis was performed according to Lu *et al.* [25]. Genomic DNA was extracted with Fast DNA Spin for soil kit. 16S rRNA were amplified by primer set 63F (5ʹ-CAG GCC TAA CAC ATG CAA GTC-3ʹ) [26] with a fluorescent label on the 5 prime end, and 1389R (5ʹ-ACG GGC GGT GTG TACAAG-3ʹ) [26] and PCR products were digested with the restriction enzymes (RE) *MspI* (5ʹ-C^C G G-3ʹ) (ThermoFisher Scientific, Waltham, MA, USA) for terminal restriction fragments length polymorphism (TRFLP) analysis.

## 3. Results 

### 3.1. Major VFA Products

Yields per mole of consumed glucose were calculated for acetate, butyrate, ethanol, propionate, lactate, succinate, hydrogen, formate, and biomass. Glucose was always completely consumed within the margin of error in the COD balance (<10%), and all balances closed within 10%. Glucose was only found in the effluent during the reset pH approach between pH 6.5 and 7.5 (23%–25% glucose was not consumed). Products such as valerate, hexanoate, and pyruvate are not reported as the total of these yields was less than 0.2% of consumed glucose under all conditions.

**Figure 2:**
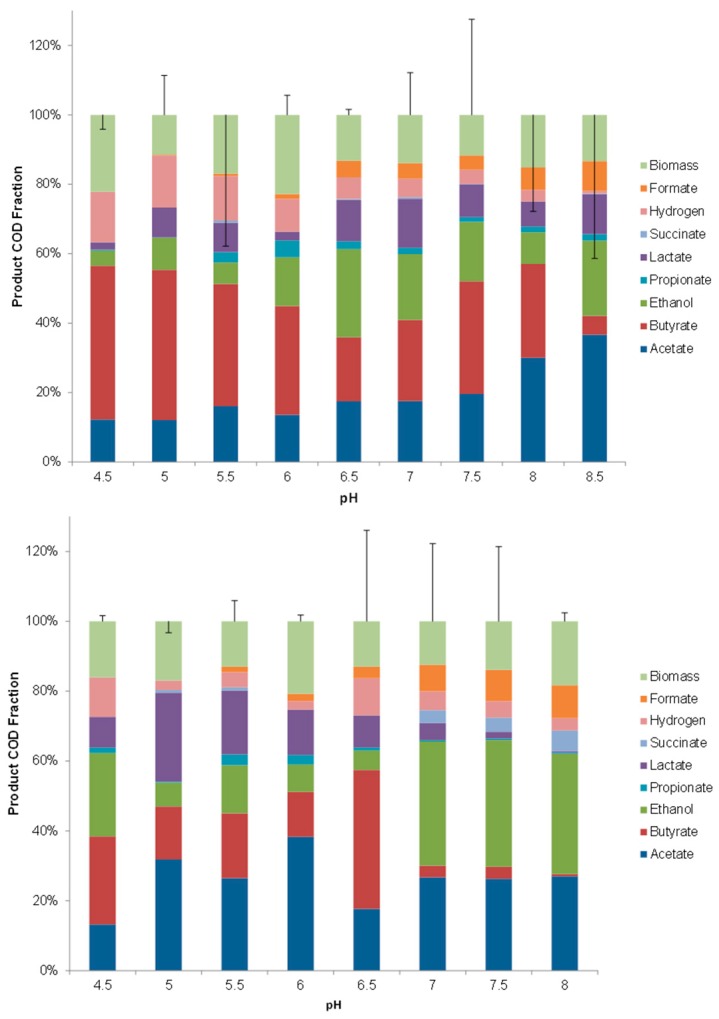
Influence of different pH regulation approach to product spectrums of glucose (in % produce) fermentation in progressive pH approach (top) and reset pH approach (bottom). Error bars are 95% confidence intervals based on two-tailed *t*-tests (*n* = 5).

**Figure 3:**
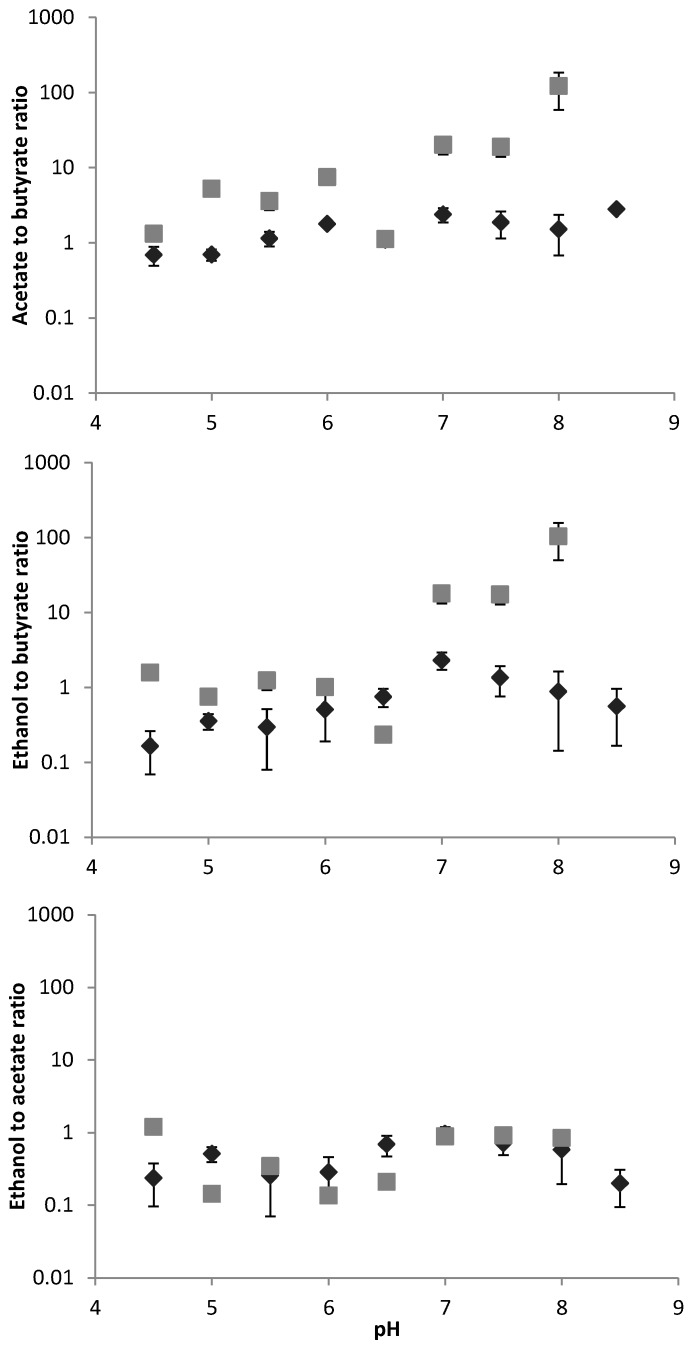
Influence of pH regulation approach on relative acetate:butyrate, ethanol:butyrate, and ethanol:acetate ratios for reset pH approach (grey square) and progressive pH approach (dark diamond) (Note: logarithmic scale).

Figure 2 shows the effect of pH on the product yields in mixed culture glucose fermentation. The error bars indicate the error in the COD balance of the inlet substrate and the total COD yield at the outlet. In general, errors were smaller for reset pH approach compared to progressive pH approach, partly because of variations between the two progressive pH approach runs, and partly because of improved methods during reset pH approach (it was the last experiment done). The fractional yield of each product (shown in Figure 2) indicates (from bottom to top) that while spectrum was broad, acetate, butyrate, and ethanol were the dominant products (60% of total product, 80% of catabolic product). In general, there was a similar trend with a shift from butyrate to ethanol/acetate as pH increased, but the two pH regulation methods resulted in very different shift patterns. Specifically, progressive pH variation caused a gradual shift from butyrate-acetate to acetate-ethanol as pH increased, while reset pH variation caused a step change from acetate-butyrate, to acetate-ethanol fermentation above pH 6.5. Ethanol to acetate ratios were not substantially influenced by pH (Figure 2), while ethanol:butyrate and acetate:butyrate were significantly and similarly affected (Figure 3). This indicates a bimodal system, with either butyrate dominant fermentation, or ethanol-acetate dominant fermentation. The reset pH approach resulted in a clear product shift at pH 7, particularly with an almost complete shift to ethanol-acetate fermentation (Figure 3).

Of the C_3_ products, only lactate appeared in substantial amounts, and largely during reset pH approach at low pH values. In both cases, biomass yield was maximized at pH 6, but overall, biomass was not substantially influenced by pH, averaging 20% (Figure 2).

### 3.2. Hydrogen and Formate (Electron Sinks)

**Figure 4:**
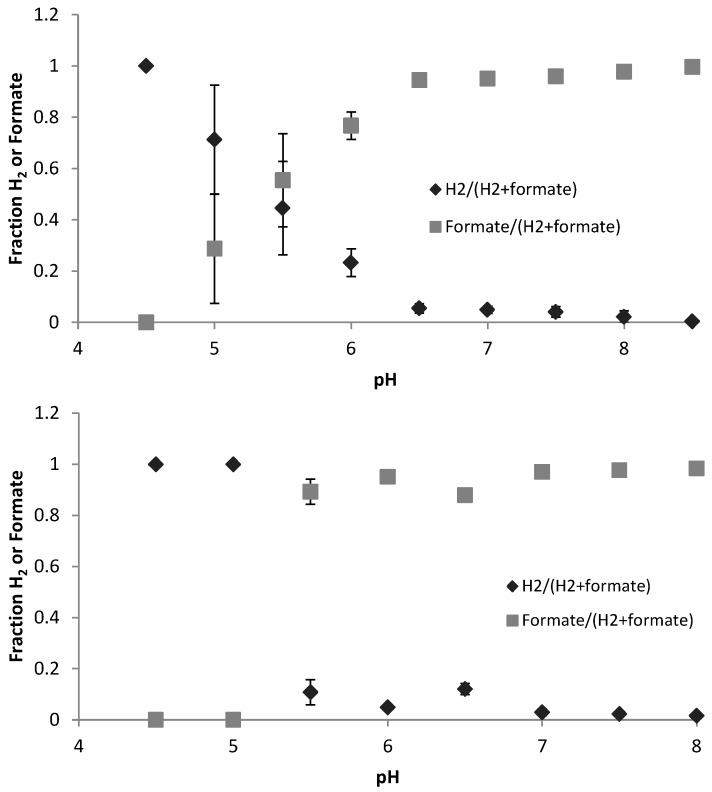
Influence of pH regulation approach on the hydrogen and formate produced as a function of pH in progressive pH approach (top) and reset pH approach (bottom). Ratio of H_2_ to total H_2_ and formate is indicated as dark diamond and ratio of formate to total of H_2_ and formate is indicated as grey square.

Hydrogen and formate are important as default electron sinks. Hydrogen and formate showed a similar trend for the different pH regulation methods from low to high pH, with mainly hydrogen being produced at low pH, and mainly formate at high pH. However, the reset pH approach showed a greater degree of response, while for progressive pH approach a gradual change with pH was observed (Figure 4).

### 3.3. Microbial Community 

**Figure 5:**
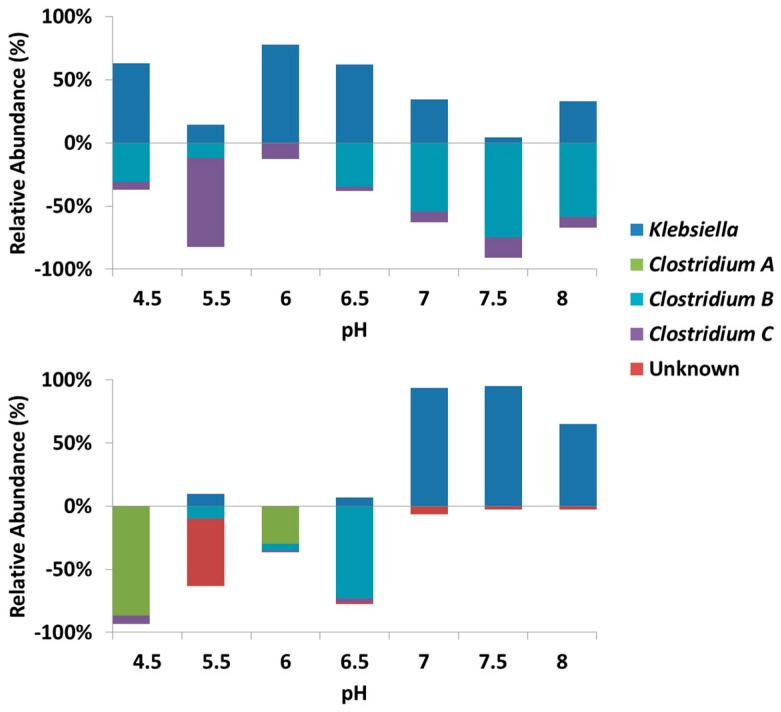
Species affiliated T-RFs (16S rRNA gene target) proportional area for progressive pH approach (top) and reset pH approach (bottom) at each pH point. Bars are split based on *Klebsiella* (top) and *Clostridium* and unknown (bottom) prominence. *Klebsiella* was identified as *K. rennanqilfy*, *Clostridium* A was identified as *C. thermocellum*, *Clostridium* B was identified as *C. botulinum* and *intestinale*, and *Clostridium* C was identified as *C. pasteurianum*.

The two pH control approaches resulted in a different microbial response to pH changes (Figure 5). The TRFLP analysis used generally identified the majority of the community, with five major peaks except pH 6 in the reset pH approach, which had a much more diverse community. pH 5.5 was the starting point of both approaches, however, progressive and reset pH approach were dominated by *Clostridium C* and Unknown T-RFs respectively. Despite the microbial community at pH 5.5, *Klebsiella* dominated at low pH ranging from 4.5 to 6.5 for progressive pH approach with *Clostridium B* emerging at pH 6.5 and dominating after pH 7. The opposite trend was observed in the reset pH approach where *Klebsiella* dominated at high pH ranging from 7 to 8, while *Clostridium A* and *B* dominated at pH 4.5 and 6.5, respectively.

## 4. Discussion 

As pH was progressively changed, the product spectrum also gradually changed. This is consistent with our previous findings where the fermenting culture retains some degree of function from the previous set point [25]. This can be partially explained by the gradual adaptation of the culture to the changing pH [20]. In the reset pH experiment, however, the product spectrum suddenly swaps from acetate-butyrate production to acetate-ethanol production from pH 6.5 to 7. One possible explanation is that the culture community changed from acetate-butyrate to acetate-ethanol producers.

As identified in previous work [25] on the reset pH approach, there is generally a carry-over of the microbial population from the preceding pH to the subsequent pH (high or low). In addition, the speed of phylogenetic changes in response to the environment is slower than chemical changes (*i.e.*, species present at central pH points of 5.5 were also present at subsequent stages). This may explain the variation of microbial community at the same pH through different approaches, especially on *Klebsiella* and *Clostridium*. While genus *Clostridium* is commonly associated with acetate and butyrate production from glucose [26], there is some discrepancy between this expected phenotype and the T-RFs that were identified. *C. thermocellum* was closely related to *Clostridium A* T-RF [25] dominating at pH 4.5 and 6.0 with high acetate production in reset pH approach. However, *C. thermocellum* has been studied primarily for ethanol and acetate production at neutral pH, and generally under thermophilic conditions [27]. Similarly, both *C. botulinum* and *C. intestinale* were closely related to *Clostridium B* T-RF [25] dominating at pH 6.5 or 7.0–8.0 in reset and progressive pH approaches, respectively, where butyrate production increased. The generally expected product profiles for these species are butyrate, acetate, and lactate under acidic and under neutral pH conditions [28]. Interestingly, butyrate was the major product at pH 4–6 in progressive pH approach (Figure 4) which seems to be mainly contributed by *Clostridium B* and *Clostridium C* (closely related to *C. pasteurianum,* [13]), although *Klebsiella* T-RF dominated. *Klebsiella* is generally associated with 2,3-butanediol and acetoin production [29]. However, *K. aerogenes* (*K. mobilis*) has been shown to produce ethanol, acetate, and formate in glucose-limited conditions that were similar to this study [30]. Sequences of *Klebsiella* T-RF were obtained from the clone library [25] and were placed within unclassified *Klebsiella*, although the closely related identified group is *K. planticola* which produces formate and ethanol from fermenting glycerol [31].

There is a clear split in reset pH approach between the ecology at pH ≤ 6.5 and pH ≥ 7 from *Clostridium* shifting to *Klebsiella* while the opposite was identified in the progressive pH approach. These trends reflect the product spectrums of each approach and suggest that the act of resetting the pH enables the switch from a culture of acetate-butyrate producers to a culture of acetate-ethanol producers. However, the mechanism enabling this switch is unclear. Resetting the pH may either stress *Clostridium*, or strengthen *Klebsiella*, allowing for equal contention at the following pH. It is also unclear whether resetting to an alternative pH would provide the same effect, or if this is only seen when resetting to an acidic pH.

The order of pH progression in the progressive pH approach likely played a role in the observed product spectrum. It is unknown whether in a scheme running from high pH to low, high ethanol production would have persisted well into the acid range. The high butyrate and low ethanol production at low pH (immediately after the high range in the experimental progression) could be due to resetting the pH to 5.5 following 8 allowed for re-emergence of the acetate-butyrate metabolic characteristic.

The reset pH approach resulted in a more selective and comparable product spectra than the progressive pH approach, likely due to a lesser bias (towards *Klebsiella* as described above). Temudo *et al.* [12] suggests that the response of microbial community is strongly dependent on the cultivation history. This work demonstrates that it is the discreteness of metabolic shift, rather than the general outcomes which are influenced by cultivation history. That is, the same products result, but that the culture is more responsive when conditioned by a dynamic pH regime.

**Figure 6:**
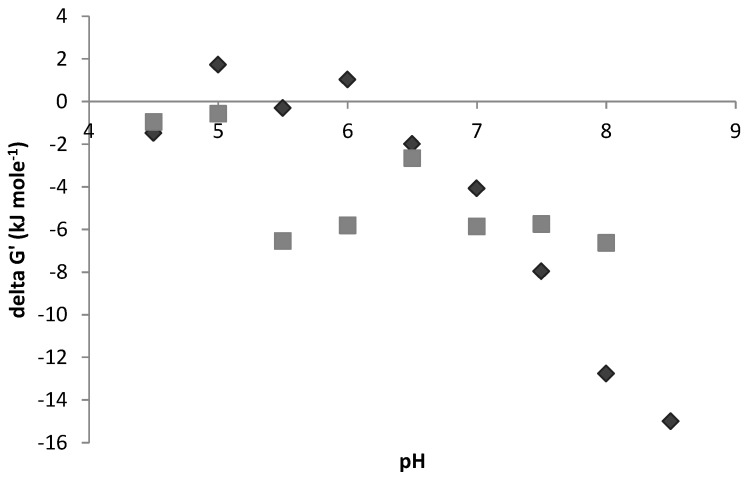
Gibbs free energy (kJ/mole) for the reaction CO_2_ + H_2_ ↔ HCOOH of reset pH approach (grey square) and progressive pH approach (dark diamond). Equation ΔG’ = ΔG° + RT ln Q, where ΔG° = ΔH° − ΔS° is used for calculation.

Figure 4 indicates that the shift from hydrogen to formate also follows this trend. Further analysis can be done on the hydrogen-formate subsystem by calculating the distance from thermodynamic equilibrium (ΔG’ = 0) for the reaction HCOOH ↔ H_2_ + CO_2_ (Figure 6). A more detailed analysis of this in context of fermentation as a whole can be found in González-Cabaleiro *et al.* [6]. Points above the line mean more formate is produced than is thermodynamically favorable, points below the line mean more H_2_ is produced than is thermodynamically favorable. The results indicate that H_2_ was generally favored as an electron sink over formate. The results from the progressive pH approach show a greater deviation toward excessive H_2_ production at higher pH than in the reset pH approach, supporting the assertion that the culture is retaining previous function. Additionally, as discussed by Hoelzle *et al.* [7], H_2_ versus formate production is determined, at least partially, by the pH-dependent enzyme formate dehydrogenase (Fdh), and by the relative expression of two different pyruvate conversion pathways. Comparing the persistent production of H_2_ at the expense of formate at high pH in the progressive approach with the sudden swap in production in the reset approach, it is suggested that Fdh may remain active when the change in pH is not drastic.

Even though both regulation approaches had a similar overall product spectrum, relative fractions of some particular intermediates, such as lactate, were different. According to Madigan *et al.* [8] there are two main factors affecting fermentation type, including (1) accumulated mass of fermentation products in the reactor, and (2) ratio of NADH/NAD^+^ inside the microbial cells. NAD^+^ is the principle intracellular electron acceptors and the ratio of NADH/NAD^+^ determines the intracellular redox state. NADH is oxidized to NAD^+^ by reduction of hydrogen ions to hydrogen gas. According to Temudo *et al.* [5], hydrogen production is independent of NADH generated during glycolysis but extensively utilizes NADH from pyruvate oxidation to acetyl-CoA. Therefore, where NAD^+^ cannot effectively be regenerated by production of hydrogen gas or formate, lactate (or other reduced intermediates, including ethanol) may accumulate. Hydrogen as terminal electron acceptor becomes less favorable at increased pH due to a lack of hydrogen ions, and formate is instead favored. The more rapid shift as observed in reset pH approach can enable organisms to better utilize different pathways to use either hydrogen or formate as terminal electron acceptor. 

## 5. Conclusions 

Production of fermentation products was affected by both specific pH set-point and the way pH was varied. Although a common product spectrum (acetate, butyrate, ethanol) was observed in all pHs of both approaches, a shift was observed from butyrate at <pH 6.5 to ethanol at >6.5. Applying “jumps” in regulating pH allows the system to become more responsive. This is also shown by microbial analysis, which was more dynamic under reset conditions, and bias towards *Clostridium* spp. at low pH. Analysis of Gibbs free energy indicates that when pH was reset to a common value of 5.5 between experiments, the system operated closer to thermodynamic equilibrium than when pH was progressively changed from low to high values (and vice versa). Thus reset pH method is more stable and allows product selection. 
